# Health equilibrium in Korean adults with type 2 diabetes mellitus: A hybrid concept analysis

**DOI:** 10.1002/nop2.1593

**Published:** 2023-01-19

**Authors:** Kawoun Seo, Youngshin Song

**Affiliations:** ^1^ Joongbu University Department of Nursing Chungnam Korea; ^2^ Chungnam National University College of Nursing Daejeon Korea

**Keywords:** adaptation, concept analysis, diabetes mellitus, health, nursing

## Abstract

**Aim:**

The aim of the study was to provide a concept analysis of health equilibrium among patients with diabetes and introduced its operational definition.

**Design:**

A concept analysis was conducted using a hybrid model of Schwartz‐Barcott and Kim (Nursing research methodology: issues and implementations, Aspen, 1986).

**Methods:**

Using consolidated criteria for reporting qualitative research guidelines, 10 participants with diabetes mellitus were interviewed. Each participant conducted at least two interviews, with each interview session lasting approximately 20–60 min at home or in a quiet place with some privacy. Data were analysed using a grounded theory approach.

**Results:**

The health equilibrium concept included four categories with 12 attributes: cognitive (commitment to health, willingness to make life adjustments, balanced awareness, maintaining control), social (social role performance, holding a social support system, participation in social relationships), behavioural (leading a balanced life, making efforts to maintain health, modulating overreaction diabetes) and psychological (hopefulness for a healthy life psychological stability) factors. Thus, health equilibrium was defined as a state in which remain committed to health, while maintaining a stable daily life, social relationships and psychological stability despite prejudices against the disease and self‐care experiences.

**Conclusion:**

Health equilibrium for diabetes patients was defined as maintaining cognitive, social, behavioural and psychological equilibrium as a process of willingly adjusting to life with diabetes. This can help people with diabetes improve self‐care and maintain social roles.

## 1 INTRODUCTION

Strict self‐care is essential in managing type 2 Diabetes Mellitus (DM) (Elnaggar et al., 2020). For example, complying with a strict diet that involves proper amounts of food; restriction of monosaccharides; and consumption of a high‐protein, low‐carbohydrate diet is crucial (International Diabetes Federation, 2021). Complying with the recommended diet often requires individuals to avoid dining out and consume only certain types of foods when eating with other people, which may prove challenging (Seo & Song, 2019). Because DM is perceived to be caused by a poor lifestyle, individuals with DM find it difficult to disclose their condition and instead continue consuming a regular diet enjoyed by non‐DM populations rather than adhering to their recommended diet (Yoo, 2017). Furthermore, they avoid social gatherings or dining out with other people, which hinders their social relationships (Seo & Song, 2019). This is perhaps owing to a problematic re‐identification process in which individuals perceive themselves as a “diseased person” after the onset of DM (Larsen, 2017). If this leads to social intimidation or giving up on one's self‐care, it can lead to a deteriorated quality of life (Nam et al., 2013; Seo & Song, 2019). Health equilibrium refers to a state of maintaining a balance between the internal state and the external state, including social relationships, to improve the quality of life in order to maintain health. After being diagnosed with diabetes, some diabetes patients are often found to be unable to balance their daily activities, including diabetes self‐care and social activities. Yet other diabetes may begin to embrace their diabetes and see improvements in quality of life by balancing diabetes self‐care and daily living. As such, if a diabetes patient goes through the process of re‐recognition as having a disease, he or she will accept diabetes and have a balanced life between disease management behaviour and social behaviour. However, until now, the concept of quality of life including physical, mental, psychological and social factors of diabetes patients is absent.

## 2 BACKGROUND

Many studies that examined the disease experiences of patients with DM documented that these individuals accepted and adjusted to their condition and moved on with their lives (Sebire et al., 2018; Stuckey & Peyrot, 2020; Yi et al., 2014; Yoo, 2017). Patients with DM may accept DM as an inevitable, painful companion for the rest of their lives; adjust their daily lives, accordingly; disclose their condition to others; and learn treatment methods that suit them after trial and error (Sebire et al., 2018; Yi et al., 2014). Furthermore, they strive to convert their weaknesses into opportunities and change the direction of their lives (Kim, 2017). As shown here, there are patients with DM who overcome the negative aspects of the disease and maintain or even improve their quality of life, describing themselves as healthier than before (Yoo, 2017). In fact, in previous study that reviewed at qualitative research on diabetes patients, there were negative adaptations to the experience of living as a diabetes, but a positive adaptation, that is acceptance of diabetes and integration into life (Stuckey & Peyrot, 2020).

It is difficult to apply a dichotomous classification (healthy or unhealthy) to patients with lifestyle‐related diseases, such as DM. In particular, it is inappropriate to refer to diabetes patients who live at home, which is their home away from the hospital, just because they have the disease. If their disease is well controlled, it will not be evident to others; thus, they may not perceive themselves as patients, continuing to carry on with life as “normal” (Nam et al., 2013). Thus, their health should be viewed as a continuous construct for which a dynamic equilibrium between internal and external environments should be attained (Kim, 2012). Such a dynamic balance of health status is known as health equilibrium.

In South Korea, the concept of health equilibrium has been analysed among older adults with chronic diseases (Kim, 2012). This study defined equilibrium as a balance of health between the internal and external environments achieved by overcoming a chronic disease based on health awareness, determination and mobility from a family friendly dimension, and expansion of relationships and networks and engagement in active interactions from a social relationship‐oriented dimension (Kim, 2012). However, this definition cannot be directly applied to patients with DM, as the concept was analysed among older adult populations and its attributes are limited to social relationships. Also it is difficult to find a study analysing the concept of “health equilibrium” in other countries. So far, the concept of health‐related equilibrium has been mentioned in relation to health insurance.

When patients with DM go through the process of re‐recognition of the diseased individual (Larsen, 2017), it is thought that self‐stigma will decrease, and they will have a balanced life between disease management behaviour and social behaviour. However, up to now, the concept of quality of life including physical, mental, psychological and social aspects of patients with DM is lacking. Considering the stable and high quality of life of some patients with DM, it is important to understand their perceived health equilibrium and to provide an opportunity for patients with DM in South Korea to reflect on their perceptions about their disease (Seo, 2021; Yoo, 2017). In addition, the concept of health equilibrium in patients with DM is clarified to help understand those who are not faithful to self‐care or suffer from withdrawal of social relations. The definitions, attributes and indicators extracted as a result of this study can contribute to the development of instruments that can objectively measure their quality of life.

In this context, we analysed the concept of health equilibrium in patients with DM using a hybrid model to identify and analyse the domains, attributes and indicators of “health equilibrium” and define the concept.

## 3 METHOD

### 3.1 Study design

This study is a concept analysis of health equilibrium in patients with DM. We extracted the domains, attributes and indicators of the concept through three phases (theoretical, fieldwork, and final analytical) proposed by the hybrid model (Schwartz‐Barcott & Kim, 1986). The hybrid method (Schwartz‐Barcott & Kim, 1986) is an effective strategy because it explores real‐world phenomena based on a rigorous literature review.

The theoretical phase consisted of the selection of concepts, a literature review, exploration of meaning and measurement and selection of operational definitions (Schwartz‐Barcott & Kim, 1986). This initial phase involved the selection of the concept of interest and a review of relevant literature to clarify the operational definition of the concept to be used in the subsequent fieldwork phase. In this phase, the definitions, attributes, antecedents and consequences of the concept were identified. We reviewed the dictionary definition and relevant literature to define the concept of health equilibrium in patients with DM and identify its domains and attributes.

The fieldwork phase is when the domains and attributes of the concept discovered through the literature review in the previous theoretical phase are examined and expanded. The contents analysed in the theoretical phase are confirmed through interviews and observations in the field, in which the actual phenomenon can be observed, and the collected data are analysed and integrated with the results of the theoretical phase.

In the final analytical phase, the researchers integrated and analysed the literature analysis data and fieldwork data through discussion to confirm the definition, attributes and indicators of health equilibrium in patients with DM.

### 3.2 Search strategy

**FIGURE 1:**
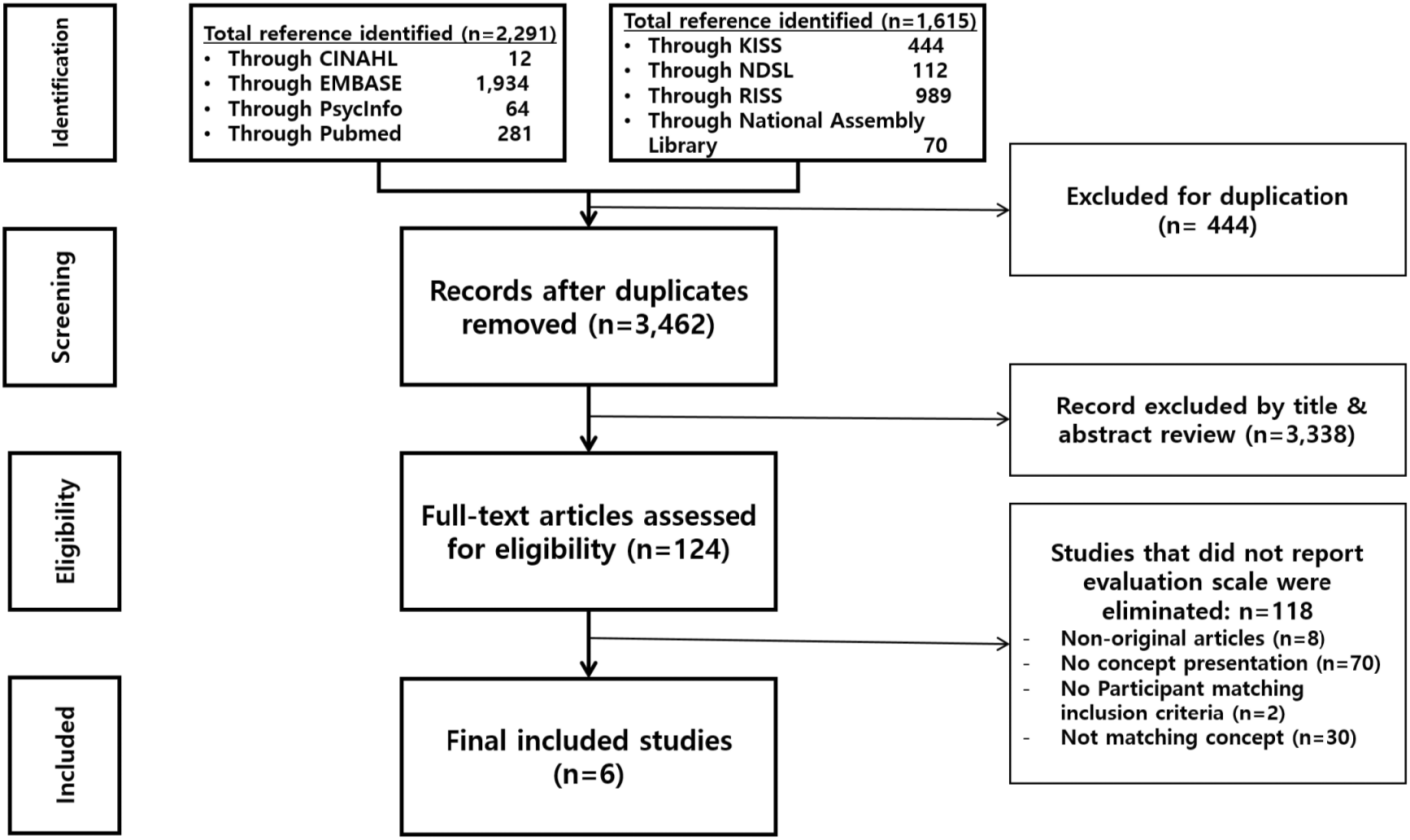
Search strategies for the theoretical phase

Foreign literature was searched in PubMed, EMBASE, PsycINFO and CINAHL using a combination of terms: health, equilibrium, normality and normalcy. Korean literature was searched in the Research Information Sharing Service, Korean Studies Information Service System, National Digital Science Library and National Assembly Library without limiting the year of publication using the terms health and equilibrium in the title, keywords or text. The search strategies are shown in Figure 1. A total of 3809 search results were generated, and the final six studies were selected. Table 1 lists the attributes of these six studies.

**TABLE 1:** Literature on the attributes of health equilibrium in patients with diabetes

Author(s)	Attributes
Headey and Wearing (1989)	Normal pattern of life events, level of subjective well‐being, predictable basis of stable person characteristics
Yurkovich and Lattergrass (2008)	In balance or a sense of harmony, having equilibrium and not being out of control of their being, which includes spiritual, cognitive, emotional and physical domains; empowered through knowledge of illness, and/or honest self‐awareness; maintaining a sense of control over their well‐being and symptoms while having hope; formal healthcare system (mental health providers) and informal networks (family, friends and peers) as support resources
Kim (2008)	Includes physical, emotional, social, intellectual and psychological components; happiness; quality of life; disease treatment; disease prevention; optimal health status
Lipworth et al. (2011)	Balancing accommodation of illness against the desire to maintain some degree of normality, role fulfilment and personal control; maintaining other social roles; preserving a normal relationship; balance between preoccupation with illness and denial; balanced emotions; manage ambivalent feelings; maintain hope; avoid being overwhelmed; balanced acts of communication (pass on information without causing alarm or to acknowledge both their own expertise and that of others)
Kim (2011)	Gaining information and knowledge about the disease; aspiration and willingness to regain health with awareness of one's own self; pursuing both internal and external health at the same time; participation in health programmes; diversification of life patterns; active health management in social networks; efforts to overcome chronic diseases; initiates self‐care for better health, builds social networks; restores confidence in life; restores relationship‐oriented life; stays in an inward‐oriented relationship; tries to take good care of himself/herself; sticks to a family‐friendly life
Kim (2012)	Family friendly (is perceptive and has willpower, motivation, and patience); social relation‐oriented (cooperative)

### 3.3 Participants of the fieldwork

Ten patients with type 2 DM were interviewed from a hospital and public health centre in South Korea. The general and diabetes‐related characteristics of participants are presented in Table 2.

**TABLE 2:** Participants' demographics (*n* = 10)

Characteristic	Category	*n* (%) or M ± SD
Sex	Male	3 (30.0%)
	Female	7 (70.0%)
Age (years)	<60	3 (30.0%)
	60–69	6 (60.0%)
	≥70	1 (10.0%)
Marital status	Married	9 (90.0%)
	Not married	1 (10.0%)
Education level	Middle school	3 (30.0%)
	High school	4 (40.0%)
	University	3 (30.0%)
Job status	Employed	5 (50.0%)
	Not employed	5 (50.0%)
Subjective health status	Good	2 (20.0%)
	Moderate	4 (40.0%)
	Bad	4 (40.0%)
Duration of diabetes (years)	1–10	4 (40.0%)
	11–20	2 (20.0%)
	21–30	2 (20.0%)
	>30	2 (20.0%)
Type of treatment	PO	6 (60.0%)
	PO + insulin	2 (20.0%)
	None	2 (20.0%)
Type of management	Diet	7 (70.0%)
	Exercise	5 (50.0%)
	Blood sugar test	9 (90.0%)
	Foot management	2 (20.0%)
	No smoking	7 (70.0%)
Type of hospital	University hospital	2 (20.0%)
	Local clinic	8 (80.0%)
Satisfaction with diabetes Management	Satisfied	6 (60.0%)
	Moderately satisfied	3 (30.0%)
	Not satisfied	1 (10.0%)

Abbreviation: PO, Per Os.

We conducted at least two (preliminary and in‐depth) face‐to‐face interviews with each participant. In the preliminary interview, each participant's experiences and difficulties with diabetes management were discussed. Ten patients displayed characteristics of health equilibrium during the theoretical phase (i.e. prioritizing health, striving for health, and high quality of life). In‐depth interviews were conducted to extract the attributes of health equilibrium. The specific inclusion criteria were patients with DM who (1) voluntarily expressed willingness to participate in the interview, (2) have no psychiatric diagnosis and (3) were cognitively capable of expressing their feelings. There were no restrictions on gender, age and duration of diagnosis. The specific exclusion criteria were (1) patients with type 1 diabetes mellitus and gestational diabetes mellitus, (2) patients with cognitive problems and (3) patients not participating in self‐care.

### 3.4 Interview

In the present study, in‐depth interviews were conducted with 10 adults diagnosed with type 2 DM to reconfirm the attributes and concept of health equilibrium. Data were collected from 1 March to 25 April 2020. The participants were informed about the study's purpose and detailed procedures, including audio‐recording of the interviews. Those who voluntarily provided written, informed consent were enrolled in the interviews. In‐depth interview techniques were employed, including the use of pre‐established questions based on the domains and attributes identified in the theoretical phase, and all interviews were conducted by the researchers. All interviews were audio recorded, and the interviewees' nonverbal behaviours during the interview were noted.

The key open‐ended questions were: “How did you feel when you were first diagnosed with diabetes?” “What has changed after the diagnosis?” “How do you manage diabetes?” “What are some of your own know‐how to manage diabetes?” Each participant engaged in at least two interviews, and each interview session lasted for about 20–60 min. The interview data collected during the fieldwork were analysed using a grounded theory approach (Hsieh & Shannon, 2005). The interview transcripts were then independently reviewed by the researchers and a nursing professor with prior experience conducting qualitative research with patients with DM, to extract key concepts and classify them. Based on these results, theoretical sampling was performed.

### 3.5 Final concept definition

In the final analytical phase, the results of the theoretical and fieldwork phases were comparatively analysed to define an integrated concept of health equilibrium (Schwartz‐Barcott & Kim, 1986). In the process, we clarified the definition of the concept by asking three questions as follows. (1) How important is the concept of health equilibrium in nursing care, and to what extent is it applied? (2) Is the select of this concept appropriate? (3) Does this concept exist in the target group as you go through each stage of the hybrid model, and how often does it appear?

These results were evaluated by three DM experts (one physician and two nurses) and two nursing professors with qualitative research experience concerning DM. They confirmed that the attributes and indicators were placed at the appropriate dimensions and were suitable for patients with diabetes.

### 3.6 Methodological rigour

To increase the reliability and validity of data analysis, four stringency evaluation criteria were considered (Lincoln & Guba, 1985). Two or more interviews were conducted to rule out the researcher's bias. In addition, two different researchers independently conducted the analysis. To reach consensus, unclear areas were discussed until they were clarified, thereby increasing credibility. To improve the transferability of the study, patients with diabetes in various stages of treatment were selected as study participants. They were actively involved in self‐care, and the prevalence period of diabetes varied. Themes were created after reviewing the transcripts to ensure dependability and confirmability. The research team members—clinical experts, qualitative research experts and nursing experts—classified the extracted themes into sub‐categories with similar contents. Subsequently, the selected theme was reconfirmed and checked until a new theme appeared. We also collected discrete data such as concepts classified in the in‐depth interviews, data from research discussions and theoretical concepts extracted from the theoretical stage and used methodological triangulation in the final stage of analysis (Lincoln & Guba, 1985). The 32‐item checklist, Consolidated Criteria for Qualitative Research Reporting (COREQ), was used to ascertain the quality of the qualitative research conducted at this stage (Tong et al., 2007; see Supporting Information File).

## 4 RESULTS

### 4.1 Theoretical phase

#### 4.1.1 Meaning of “health equilibrium” in various disciplines

The concept of “health equilibrium” has generally been used in older adults in various disciplines. In a study about physical education among older adults, Kim (2008) introduced this as a new concept signifying happiness and better quality of life produced by balanced physical, emotional, social, intellectual and mental components. In the field of nursing, Kim (2012) described the concept as a balance between the internal and external environments achieved by overcoming a chronic disease based on health awareness, determination and mobility from a family friendly dimension and expansion of relationships and networks and engagement in active interactions from a social relationship‐oriented dimension.

#### 4.1.2 Concepts related to health equilibrium

Health‐related quality of life (HRQOL) specifically refers to the physical, emotional and social aspects of quality of life that are influenced by disease or treatment (Calvert & Freemantle, 2003). HRQOL is differentiated from health equilibrium in that it mostly deals with the consequences. Coherence refers to an individual's ability to appropriately utilize and control internal resources to overcome a state of tension provoked by a tension trigger in the continuum of health and disease (Antonovsky, 1979).

#### 4.1.3 Antecedents, consequences, domains, attributes and a tentative definition of health equilibrium

Table 2 shows the antecedents, domains, attributes and consequences of health equilibrium analysed in the theoretical phase. Health equilibrium was tentatively defined as a state of striving to recover health from a diseased state, achieving psychological stability by overcoming social prejudices and self‐stigma resulting from the process of disease management, and carrying on with daily living and social life at one's own pace. The antecedents included being diagnosed with the disease and engaging in self‐care behaviours, with the consequence being an improved quality of life owing to effective management of DM, maintenance of social relationships and achievement of psychological stability.

### 4.2 Fieldwork phase

#### 4.2.1 Cognitive factors

##### Commitment to health

Being committed to health means that the participants prioritize their health above all things in life and remain committed to promoting their health, such as striving to resolve any health problems.

After being diagnosed with diabetes, I thought about what I can give up and came to the conclusion that I can give up everything except for my family and myself. So, I let go of everything that I had been grasping onto at the time and committed myself to caring for my health. (Participant 1)

##### Adjusting lifestyle with will

Adjusting lifestyle with will means that the participants are determined to manage their DM, and they engage in proper self‐care and adjust their lifestyle while overcoming social prejudice and self‐stigma. It includes adjusting to the disease by developing balanced strategies and flexibly and practically adjusting their lifestyle with a desire and volition to recover their health.

My heart sunk when I was diagnosed with diabetes. As soon as I heard that I have diabetes, I quit snacking at once. I got rid of the word snack and overeating from my life, and I came to restrict my eating tremendously. I was only able to restrict myself by thinking that I might die if I don't follow this. (omitted) But I still work; so, I still have to meet people. If I do, I ask them out for a cup of tea instead of meals. (Participant 3)

##### Balancing perceptions

Balancing perceptions means that the participants recognize and accept that they differ from people who do not have DM. It refers to awareness of others' prejudice and the effects of negatively perceived self‐worth.

Before I had it, I thought of it as bad…I thought, ‘why did that person not care for themselves and got that disease?’ But after I got it, and I began to manage diabetes, I actually got healthier. First, my weight; so, I can take out my old clothes from 40 years ago and wear them again. (omitted) I believe that you can definitely overcome diabetes if you follow the rules. (Participat 3)

##### Maintaining control

Maintaining a sense of control means that the participants are confident that they have the ability to adequately control their health and disease.

Now I have adapted, and I overeat sometimes; but I'm still confident. I now have the ability to control this. (Participant 3)

#### 4.2.2 Social factors

##### Perform social roles

Performing social roles means that the participants are performing their current social roles without restrictions owing to DM, and these roles include their roles in family and society.

I'm a housewife. I can't stop cooking because I can't eat… And if you don't use sugar, then the food is tasteless. If my kids don't eat, then I think maybe that is the reason; so, I used to cook food separately for them using sugar. (Participant 2)

##### Having a social support system

Having a social support system means that the participants have supportive resources to turn to when in need of help with managing their DM, and these resources included both formal and informal support systems.

A friend of mine is an emergency medicine professor at Chungnam National University. So, when I go abroad, and I don't feel right, I call this friend and tell him, ‘Hey I have this and that. What do you think?’ And if he tells me just enjoy your time there, then I do that; and if he tells me to come back and get tested, then I do that. (Participant 4)

##### Participating in social relationships

Participating in social relationships means that the participants take part in multidimensional social activities without any restrictions. They participate in health management programmes and actively engage in gatherings within their social networks.

I go to sports clubs and meet friends, too. That is how life should be… I travel abroad often, too. Before I go abroad, I go to my doctor and get prescriptions. (Participant 4)

#### 4.2.3 Behavioural factors

##### Maintaining a balanced life

Maintaining a stable daily life means that the participants generally maintain a stable and predictable normal life, including DM management, without any restrictions.

Nothing's changed. I go to work…I just eat three meals a day regularly and try to avoid dining out with my family a little. (Participant 1)

I exercise; so, if I don't eat, my sugars drop crazy. (omitted) But if I can't exercise for some reason, then I try to adjust. (Participant 5)

##### Practising self‐care to maintain health

Practising self‐care to maintain health means that the participants search for information about DM management, practise self‐care recommendations and comply with other disease prevention and health management recommendations.

I try to eat to help improve diabetes. I control carbs and eat mostly vegetables and some meat. I eat fish and meat. I just try to avoid carbs as much as possible. (Participant 3)

##### Modulate overreactions to DM

Modulating overreactions to DM means that the participants carry on their normal lives without restricting it because of DM, while at the same time modulating their overreactions triggered by social prejudice or self‐stigma, structuring their activities and diversifying their lifestyle.

There's nothing you can't do because of diabetes. You just have to manage it well. (omitted) It doesn't harm other people. (Participant 1)

#### 4.2.4 Psychological factors

##### Hopefulness for a healthy life

Being hopeful for a healthy life means that the participants are hopeful about enjoying a healthy life, just like others, if they manage their condition appropriately.

Well, these days, people say you live longer if you have a disease. I lost a lot of weight while managing diabetes, and I got actually healthier because I care for my health. So, I feel that I have become mentally and physically healthier. (Participant 3)

##### Psychological stability

Being psychologically stable means that the participants accept themselves as people with DM but still feel that they can continue having a happy, fun and healthy life, and they have peace of mind because of this.

I just eat without thinking. I forget about everything. And I developed a habit of forgetting. I don't think twice about bad things. Let's just forget it. Just think about today. Just think about what's coming. I just live like this. (Participant 5)

### 4.3 Final analytical phase

In the theoretical phase, 12 attributes under four domains emerged: making adjustments to overcome the disease, maintaining a balanced perception, maintaining a sense of control, performing social roles, having a social support system, participating in social relationships, patterning, practising self‐care to maintain health, modulating overreaction to DM and having a positive psychological state. However, the interviews with patients with DM revealed that the most important requirement to maintain equilibrium between DM management and quality of life was to prioritize health. Thus, “commitment to health” was added to the attributes. Moreover, “patterning,” which includes lifestyle patterns and life events, was changed to “maintaining a stable daily life without DM‐related events.” Furthermore, in the behavioural domain “modulating overreactions to DM” was added to the attributes. In the psychological domain, two attributes “hopefulness for a healthy life” and “psychological stability” emerged.

#### 4.3.1 Domains, attributes and indicators of health equilibrium in patients with DM

**FIGURE 2:**
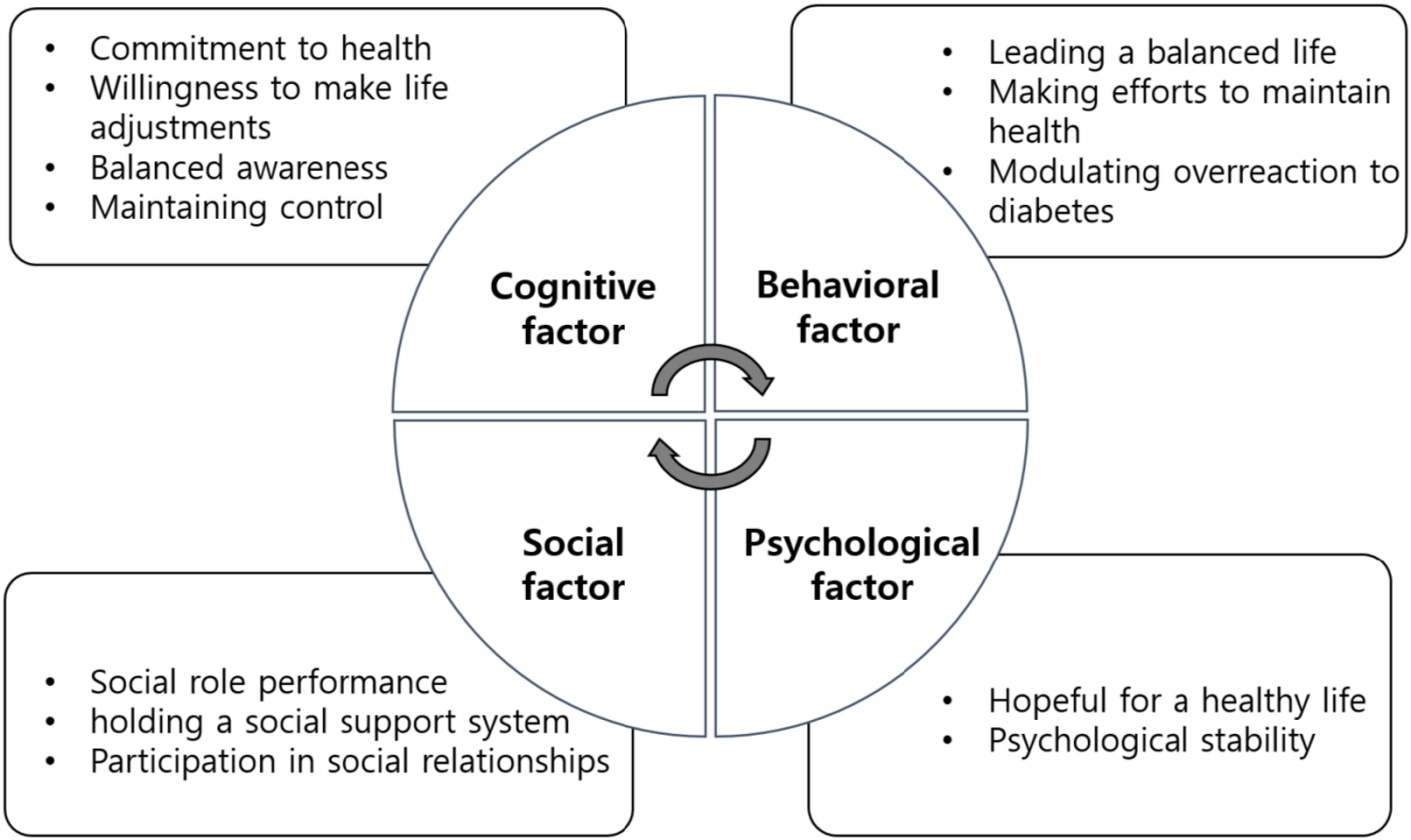
Final structure of health equilibrium for people with diabetes

During the theoretical and fieldwork phases, four dimensions, 12 attributes and 29 indicators were derived (Table 3) and the final health equilibrium attributes were determined (Figure 2).

**TABLE 3:** Domains, attributes and indicators of health equilibrium in the field study

Preceding factor	Domain	Theoretical phase			Field study	
		Attribute	Indicator	Consequence	Attribute	Indicator
Diagnosis of diabetes Diabetes management	Cognitive Factors	Adjustments to overcome Disease	Health awareness	Increased quality of life Increased psychological well‐being Confidence recovery	Commitment to health	Priority adjustment
						Immerse themselves in their health
			Inner and outer health			
					Willingness to make life adjustments	Possessing a longing and willingness to regain health
			Overcoming difficulties			
			Making efforts			
						Flexible and practical adjustment
			Strong Will			
						Balanced strategy development
			Craving a normal life			
						Adapting to the disease
		Balanced Perception	Balance		Balanced awareness	Achieving balance between sense of worthlessness and self‐worth
						Correcting prejudice and rejection of disease
			Confidence recovery			
						Awareness of oneself
			Alertness			
						Accepting the disease and minimizing its impact
		Maintain Control	Personal control		Maintaining control	Maintaining a sense of control over personal health and disease
			Disease management and control			
	Social Factors	Social role Performance	Maintaining or fulfilling social roles		Social role performance	Maintaining everyday social life
			Maintaining normal relationships			Maintaining personal roles
		Holding a social support system	Official network		Holding a social support system	Having an official health management system
			Informal network			Having an informal network as a resource for disease management support
			Building a social network			
		Participation in social roles	Participation awareness		Participation in social relationships	Participation in health programmes
			Being relationship‐oriented			Active health management in social networks
	Behavioural Factors	Patterning	Life pattern or life event		Leading a balanced life	Maintaining daily patterns such as before the disease
			Predictability			Maintaining normal daily patterns
		Making efforts to maintain health	Acquisition of knowledge and information		Making efforts to maintain health	Acquiring information and knowledge about diseases
			Learn by oneself			Maintaining normal health
			Disease management			Trying to overcome the disease
			Diversification		Modulating overreaction to diabetes	Diversification of life patterns
			Optimal			Structuring one's activities
	Psychological factors	Positive psychological state	Being hopeful		Hopefulness for a healthy life	Opportunity of improvement to an optimal level of health
			Remaining stable			Having hope
			Being happy		Psychological stability	Keep feeling happy
			Staying attached			Maintaining subjective well‐being
						Balancing attachment and separation

Cognitive factors included four related attributes (10 indicators): commitment to health, adjustment to one's life with a strong will, balanced awareness and maintaining control. Social factors included three related attributes (six indicators): social role performance, holding a social support system and participation in social relationships. Behavioural factors included three related attributes (seven indicators): lead a stable daily life, practise efforts to maintain health and control overreaction to diabetes. Psychological factors included two related attributes (five indicators): hopefulness for a healthy life and psychological stability.

#### 4.3.2 Defining health equilibrium in patients with type 2 DM

By combining the results from the theoretical phase and the fieldwork, health equilibrium for diabetes patients was defined as existing when a diabetes patient is committed to health while maintaining a stable daily life, social relationships and a stable psychological state, despite the prejudice and self‐management experience of the diabetes patient. It can be defined as maintaining cognitive, social, behavioural and psychological equilibrium as a process of willingly adjusting to life with diabetes.

## 5 DISCUSSION

The present study analysed health equilibrium in Korean patients with type 2 DM across four dimensions: cognitive, social, behavioural and psychological. Cognitive factors encompassed the cognitive aspects of maintaining health equilibrium—being committed to health, volitionally adjusting lifestyle to manage DM, striving to maintain a balanced perception and maintaining a sense of control. A prior study observed cases in which people with DM did not engage in self‐care behaviours and were satisfied with the status quo, based on the belief that they will die 1 day regardless of whether they care for DM (Mogre et al., 2019). However, most of these people regretted not committing to self‐care when they developed DM‐related complications and the symptoms were visible (Seo & Song, 2019). In the present study, the participants highlighted health as the most important factor in their lives. Although they had been shocked and fell into despair when they were first diagnosed with DM, they accepted the condition and tried to adjust over time. This is consistent with previous findings that people with DM accept their condition and adjust their everyday lives (Yi et al., 2014). Furthermore, a previous study on self‐stigma of patients with DM (Seo & Song, 2019) reported that patients with DM do not disclose their condition to other people because DM is perceived to develop as a result of a lazy lifestyle. However, our participants mentioned that they disclosed their conditions and sought help from people around them with their self‐care despite this social prejudice. Thus, people with health equilibrium strived to correct biased perceptions and maintain balance. However, we could not analyse whether this was a result of low self‐stigmatization owing to the absence of internalization of social prejudice or a result of high resilience despite having self‐stigma. Thus, further studies on the matter are required. These additional studies can be used as fundamental data when nurses reduce the disease‐related disease to patients who feel self‐stigma in clinical practice and conduct interventions that allow them to accept the disease.

The indicators of social factors were mostly about actively participating in social activities, building positive social relationships and engaging in positive interactions. This contrasted with previous reports that people with DM are socially intimidated or withdrawn owing to their self‐care efforts (Seo & Song, 2019) or that people with DM have difficulties complying with the diet recommendations because of the lack of adequate support from healthcare providers and families (Mogre et al., 2019). The study participants engaged in social activities with confidence and listed resources that could help them manage the condition. They mentioned that DM is not a barrier to their social lives, and they performed the same level of social roles as before they had DM. Some even mentioned that they developed greater confidence because they became healthier after developing DM (Nam et al., 2013). This phenomenon is the result of (Sebire et al. 2018) as well. In the Sebire et al. (2018)'s study, self‐care behaviour was initially repulsive, but as the motive was internalized over time, they rather felt pleasure in the self‐care behaviour itself. DM must be well managed to feel pleasure in self‐care behaviour and to participate in social activities and for that, a good social support system is crucial to help with self‐care activities. Thus, instead of setting DM patients as the sole target of self‐care education, education programmes should be developed for human resources such as family, friends or health professionals that can support these patients in self‐care activities.

Behavioural factors were behaviours that manifest as the participants maintain health equilibrium, and they include maintaining a stable daily life, practising self‐care to maintain health and modulating overreactions to DM. Self‐care is a lifelong project for patients with DM. Patients with DM point to being too busy, being lazy, having low access to various types of foods or diet guidelines being too restrictive as the reasons for their noncompliance with self‐care recommendations (Mogre et al., 2019). Others mentioned that they were too busy, or their surrounding environment does not help with their self‐care (Loerbroks et al., 2018). Furthermore, some stated that they eat inappropriately because they cannot disclose their condition, or they do not engage in new challenges because of their condition (Seo & Song, 2019). However, the study participants were not hesitant to practice self‐care activities for DM, and they did not hesitate to delve into social activities or new challenges. In fact, a previous study reported that people with DM acquire specific methods of DM management suitable for their own lives after numerous failures (Yi et al., 2014). Thus, understanding one's blood glucose levels and conditions is essential to promoting self‐care activities in patients with DM who have difficulties complying with self‐care recommendations. Therefore, it is necessary to understand their diet and blood sugar patterns through an individual approach to patients with DM rather than education on daily management or simply to increase the level of education and to suggest practical management methods appropriate to them.

Psychological factors refer to the psychological state that results from maintaining health equilibrium, and it encompasses the state of being hopeful for a healthy life and a stable psychological state. A study on self‐stigmatization of patients with DM reported that people with DM complained that they will die prematurely owing to DM, and that they have no hope (Seo & Song, 2019). Furthermore, patients with DM felt upset at their families for not taking care of them (Seo & Song, 2019). However, the study participants were psychologically stable and were confident that they could carry on a healthy life if they continue to manage their condition. Even though some of the participants had kidney disease as a complication of DM, had DM for more than 30 years or were receiving insulin injections, they described that their state was not different from that of other older adults. They DM in their lives, and some stated that they could manage other diseases better because they have DM. This suggests that these participants have undergone a re‐identification process—in which they have re‐identified themselves as a diseased person. This contrasts with a previous report that self‐stigmatization occurs because of the absence of re‐identification from a healthy self to a diseased self after the onset of DM (Larsen, 2017). Thus, this process of re‐identification must be understood to clarify the psychological equilibrium in patients with DM. Accordingly, subsequent studies should examine the process of re‐identification in patients with DM.

The domains, attributes and indicators of health equilibrium in patients with DM identified in this study have key implications for nursing theory, nursing education and nursing research and practice. First, the conceptual definition and specific attributes and indicators of health equilibrium established in this study are meaningful in that they provide another view for improving the quality of life of patients with DM. Second, previous studies have focused on the strict self‐care regimen that DM patients must follow; as such, their quality of life has not been described much. However, the current findings indicate that nurses should help patients change their social perception about DM. Third, currently, no instrument is available in Korea to measure health equilibrium in patients with DM. Because instruments on HRQOL or coherence have limited scope, there are limitations in interpreting the results. Developing an instrument based on the attributes and indicators identified in this study would help shed light on the quality of life of DM patients. Fourth, concerning implications for nursing practice, strict compliance with self‐care recommendations is essential to manage DM. However, examinations of patients' quality of life are lacking. Education programmes—based on the attributes and indicators of health equilibrium identified in this study that boost patients' quality of life, reduce social prejudice and promote self‐care—should be disseminated.

## 6 CONCLUSION

This study conducted a concept analysis of health equilibrium in patients with DM by exploring patients' experiences based on the results of a theoretical analysis. Based on an integration of the theoretical and fieldwork phases, health equilibrium in patients with DM was defined as a state of cognitive, social, behavioural and psychological equilibrium in which patients with DM remain committed to health while maintaining a stable daily life, social relationships and psychological stability despite prejudices against the disease and self‐care experiences. A strength of this study is that it conducted a conceptual analysis through in‐depth interviews with DM patients. However, as the study was conducted with type 2 diabetes patients in Korea, caution is needed in generalizing the results. Thus, potential cultural differences should be investigated in future studies with more expansive samples.

We expect that the concept of health equilibrium in diabetes patients outlined in this study can be used to expand nursing theory and professional education to develop a better understanding of the inclusive quality of life of this population. Current concepts can also be used to improve the current literature by developing health equilibrium scale. Finally, the findings of this study should be a useful fundament for the development of strategies to improve the quality of life in diabetes patients.

## AUTHOR CONTRIBUTIONS

K. Seo and Y. Song made substantial contributions to conception and design, or acquisition of data or analysis and interpretation of data, were involved in drafting the manuscript or revising it critically for important intellectual content, given final approval of the version to be published, and agreed to be accountable for all aspects of the work in ensuring that questions related to the accuracy or integrity of any part of the work are appropriately investigated and resolved Each author should have participated sufficiently in the work to take public responsibility for appropriate portions of the content.

## FUNDING INFORMATION

This work was supported by the National Research Foundation of Korea (NRF) grant funded by the Korea government (No. 2019R1G1A‐1,084,864).

## CONFLICT OF INTEREST

There are no conflicts of interest.

## ETHICAL STATEMENT

This study was approved by the Institutional Review Board of J University (no. JIRB‐2018090301‐01‐190,930) and conducted in accordance with recognized standards.

## Supporting information


Supporting Information File

## Data Availability

All data generated during this study are included in this published article.
