# Supplementary material for: Health equilibrium in Korean adults with type 2 diabetes mellitus: A hybrid concept analysis
Source: Nurs Open. 2023 Jan 19;10(5):3388–98. doi: 10.1002/nop2.1593 (PMC10077403; doi:10.1002/nop2.1593)
Supplement: Supplementary file 1 — Supporting Information File [file NOP2-10-3388-s001.docx]

**Consolidated criteria for reporting qualitative studies (COREQ): 32-item checklist**

| **Personal Characteristics** | **Guide questions/description** | **Study Findings** |
| --- | --- | --- |
| 1. Interviewer/Facilitator | Which author/s conducted the interview of focus group? | Dr. Seo |
| 2.Credentials | What were the researcher’s credentials? *E.g. PhD, MD* | Youngshin Song,  PhD, RN  Kawoun Seo, PhD, RN |
| 3.Qccupation | What was their occupation at the time of the study? | Professor |
| 4.Gender | Was the researcher male or female? | Female |
| 5.Experience and training | What experience or training did the researcher have? | We have taken  several classes for qualitative research courses in 2018 and  2019 in Korea. |
| **Relationship with participants** |  |  |
| 6.Relationship established | Was a relationship established prior to study commencement? | No |
| 7.Participant knowledge of the interviewer | What did the participants know about the researcher? *e.g. personal goals, reasons for doing the research* | Participant information sheet and consent form |
| 8.Interviewer characteristics | What characteristics were reported about the interviewer/facilitator? *e.g. Bias, assumptions, reasons and interests in the research topic* | Methods |
| **Theoretical framework** |  |  |
| 9.Methodological orientation and Theory | What methodological orientation was stated to underpin the study? *E.g. grounded theory, discourse analysis, ethnography, phenomenology, content analysis* | Methods |
| **Participant section** |  |  |
| 10.Sampling | How were participants selected? *e.g. purposive, convenience, consecutive snowball* | Methods (convenience) |
| 11.Method of approach | How were participants approached? *e.g. face-to-face, telephone, mail, email* | Methods (face to face) |
| 12. Sample size | How many participants were in the study? | Methods (10 participants) |
| 13. Non-participation | How many people refused to participate or dropped out? Reasons? | None |
| **Setting** |  |  |
| 14. Setting of data collection | Where was the data collected? *E.g. home, clinic, workplace* | Methods |
| 15.Presence of non-participants | Was anyone else present besides the participants and researchers? | Methods (home and office) |
| 16.Description of sample | What are the important characteristics of the sample? e*.g. demographic data, date* | Methods  (Demographics (age, gender, duration of diabetes etc.) |
| **Data collection** |  |  |
| 17.Interview guide | Were questions, prompts, guides provided by the authors? Was it pilot tested? | Methods (yes) |
| 18.Repeat interviews | Were repeat interviews carried count? If yes, how many? | Method (minimum twice) |
| 19.Audio/visual recording | Did the research use audio or visual recording to collect the data? | Method (yes) |
| 20.Field notes | Were field notes made during and/or after the interview or focus group? | Method (yes) |
| 21.Duration | What was the duration of the interviews or focus group? | Method (2 months) |
| 22.Data saturation | Was data saturation discussed? | Methods (yes) |
| 23.Transcripts returned | Were transcripts returned to participants for comment and/or correction? | Methods (yes) |
| **Data analysis** |  |  |
| 24.Number of data coders | How many data coders coded the data? | Methods (2 researchers) |
| 25.Description of the coding tree | Did authors provide a description of the coding tree? | Methods (yes) |
| 26.Derivation of themes | Were themes identified in advance or derived from the data? | Methods (yes) |
| 27. Software | What software, if applicable, was used to manage the data? | No |
| 28 Participant checking | Did participants provide feedback on the finding? | Methods (yes) |
| **Reporting** |  |  |
| 29. Quotations presented | Were participant quotations presented to illustrate the themes/finding? Was each quotation identified? *e.g. participant number* | Result (yes) |
| 30. Data and findings consistent | Was there consistency between the data presented and the findings? | Result (yes) |
| 31.Clarity of major themes | Were major themes clearly presented in the findings? | Result (yes) |
| 32.Clarity of minor themes | Is there a description of diverse cases or discussion of minor themes? | Result (yes) |
